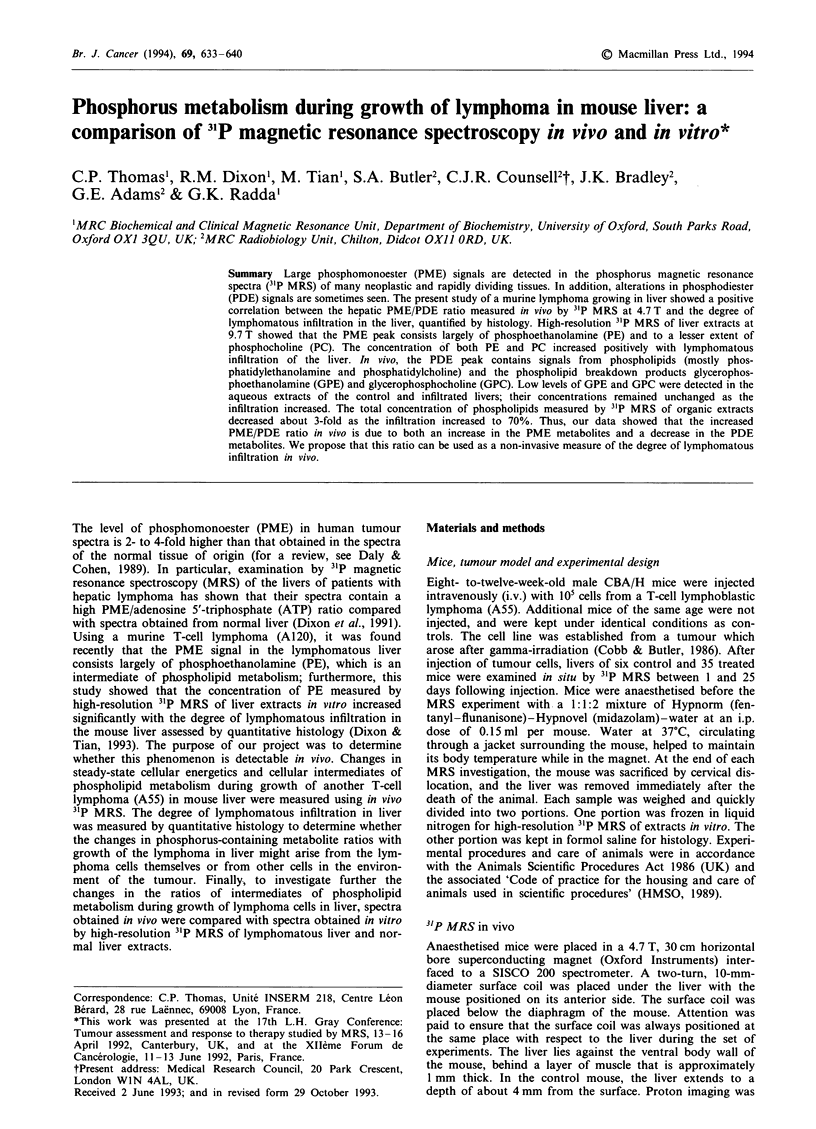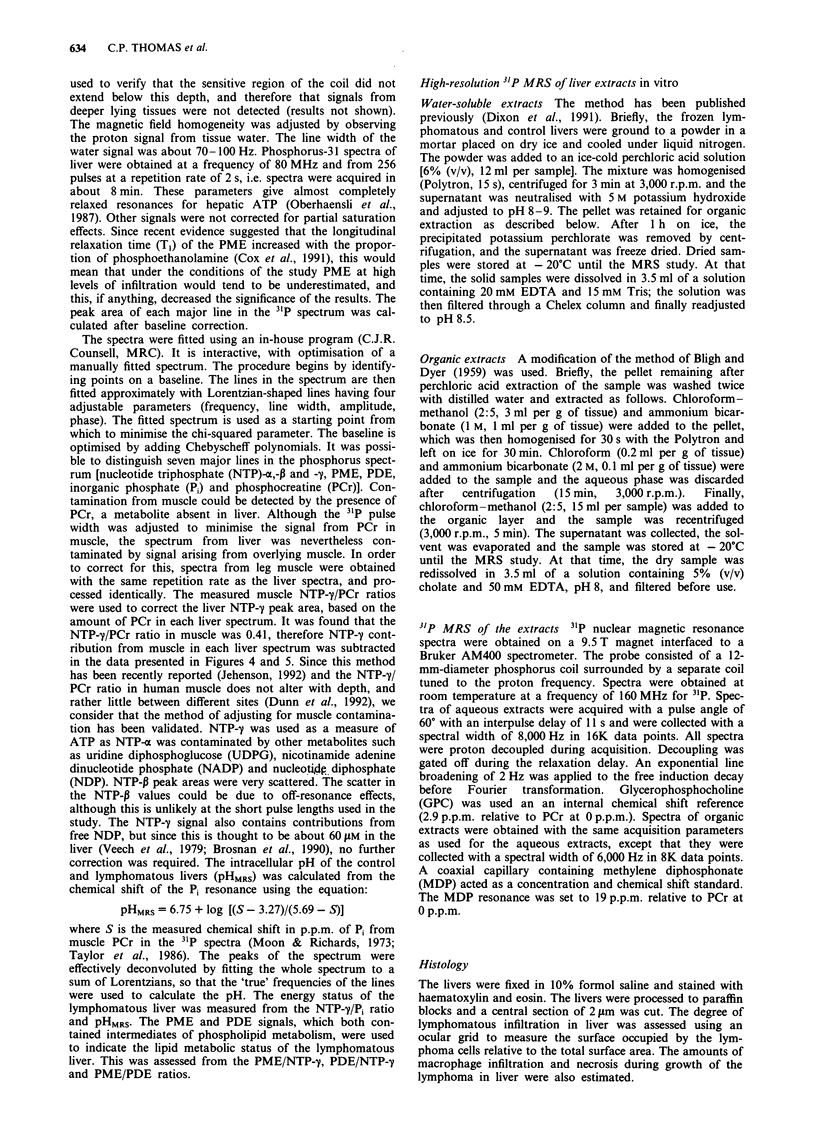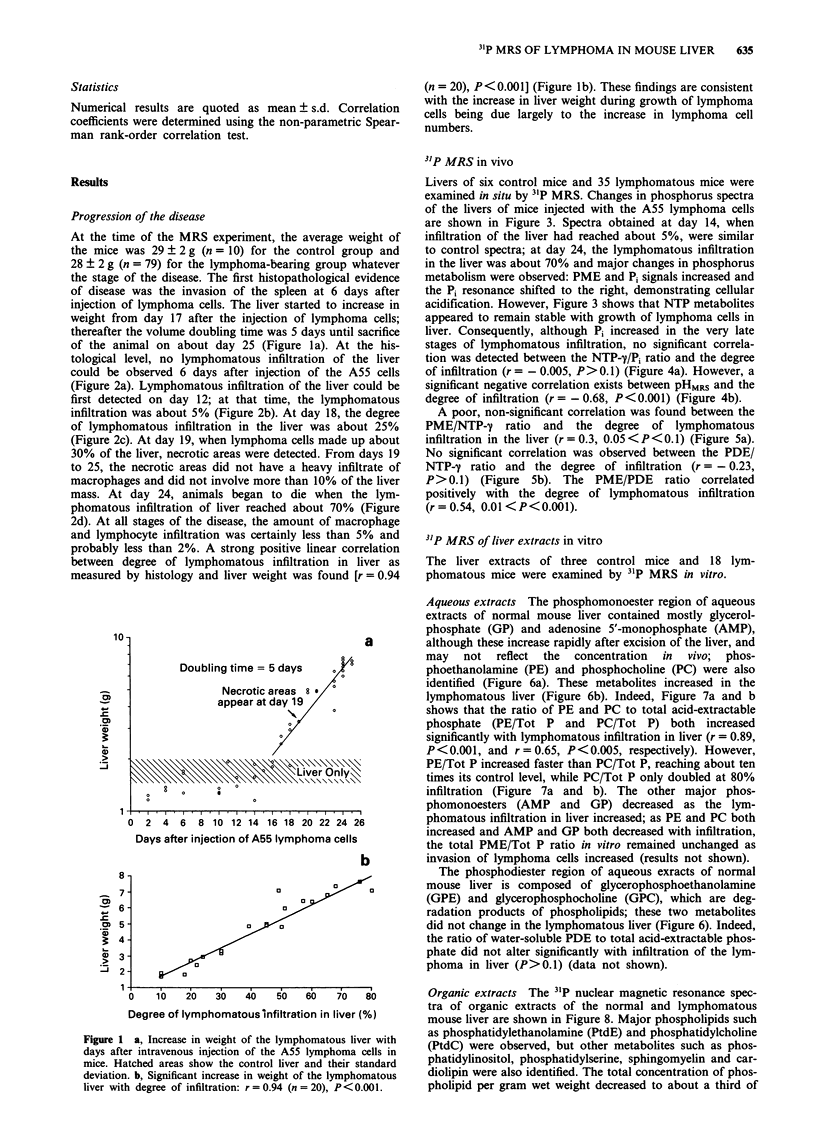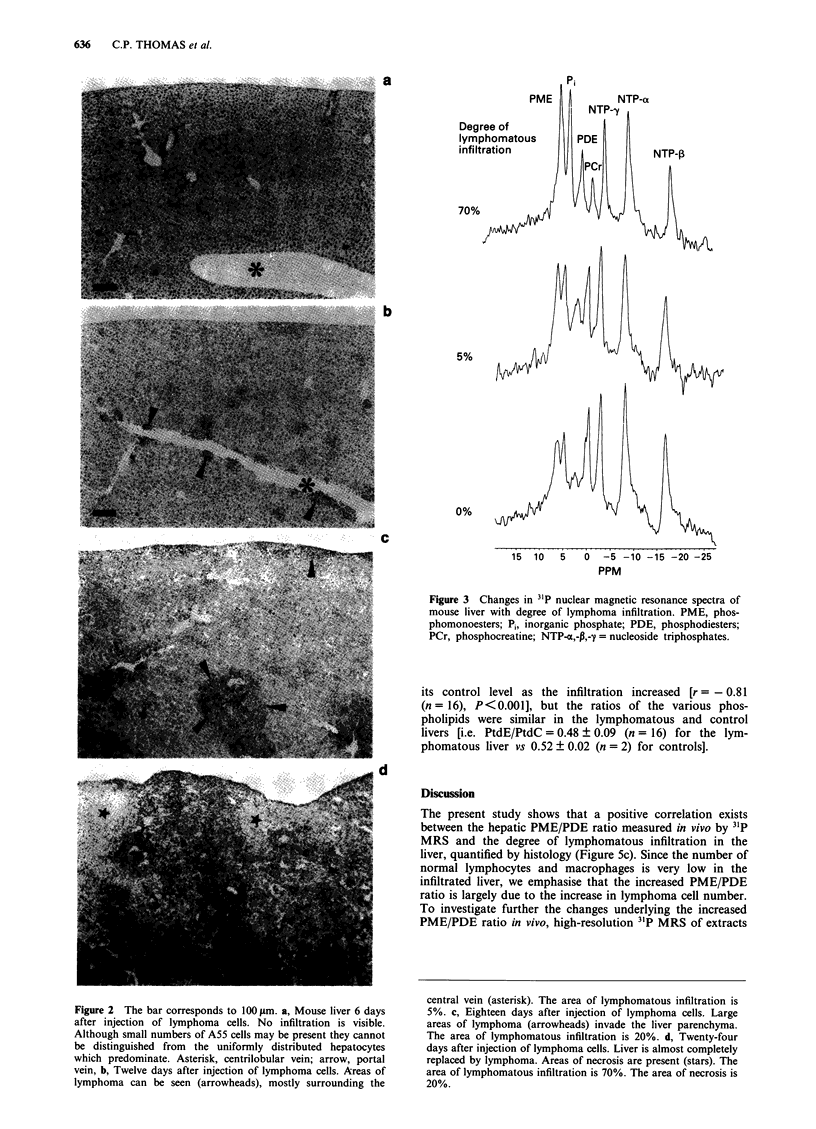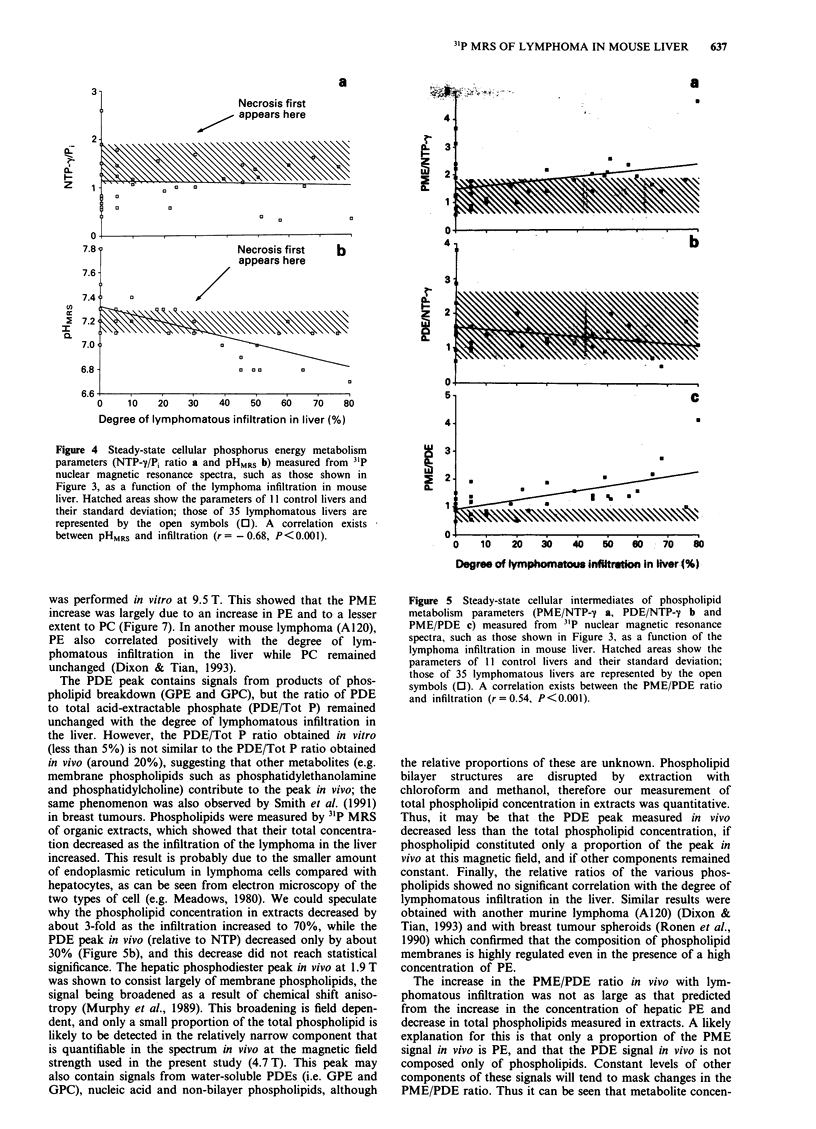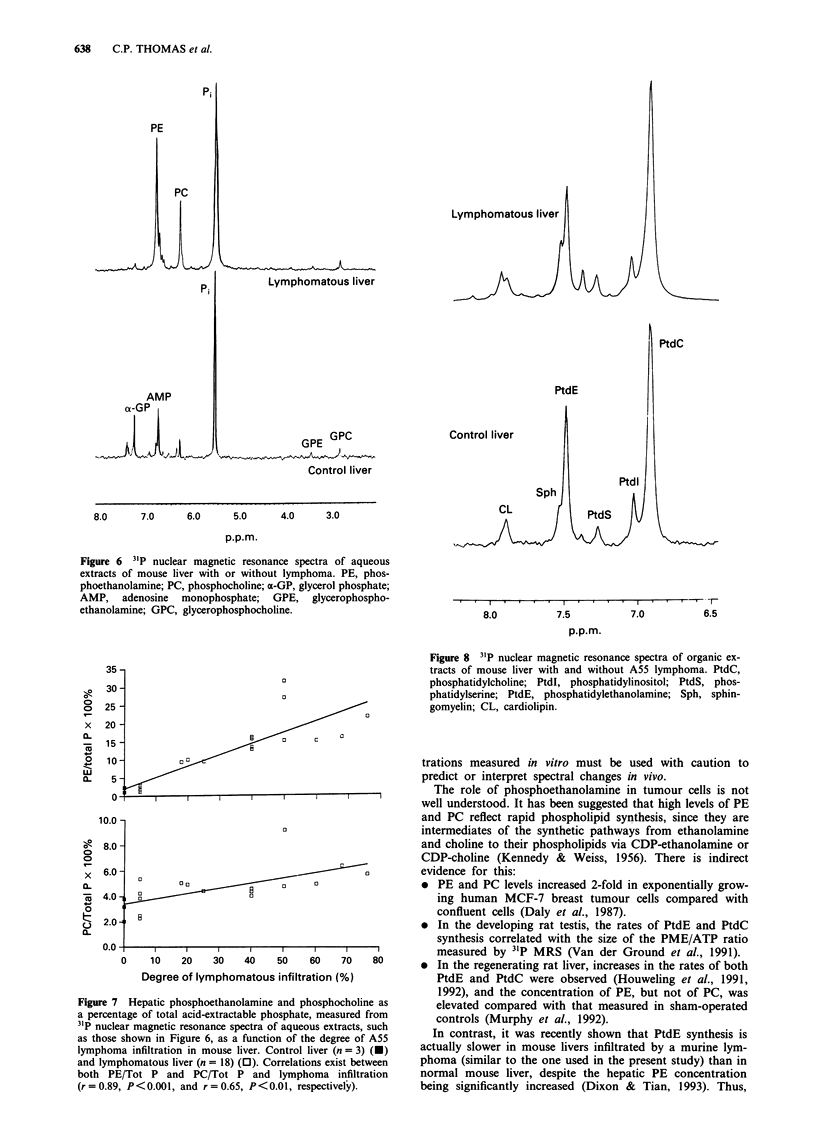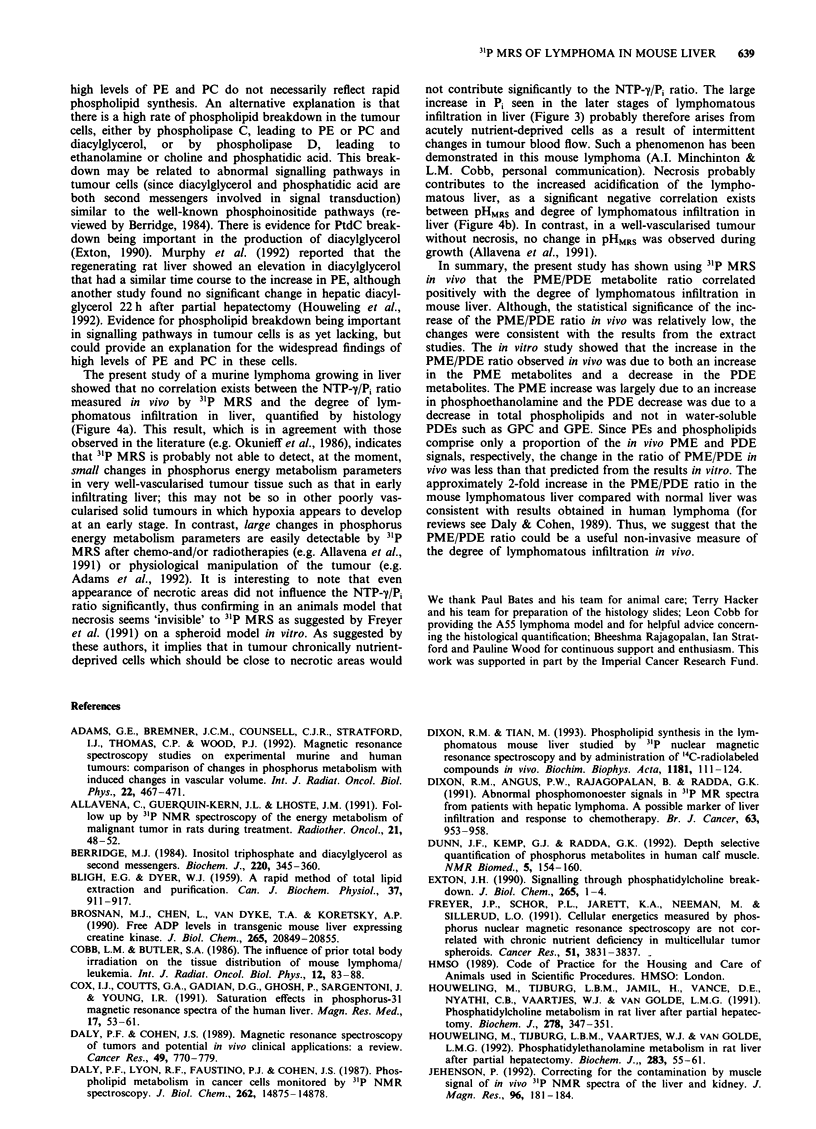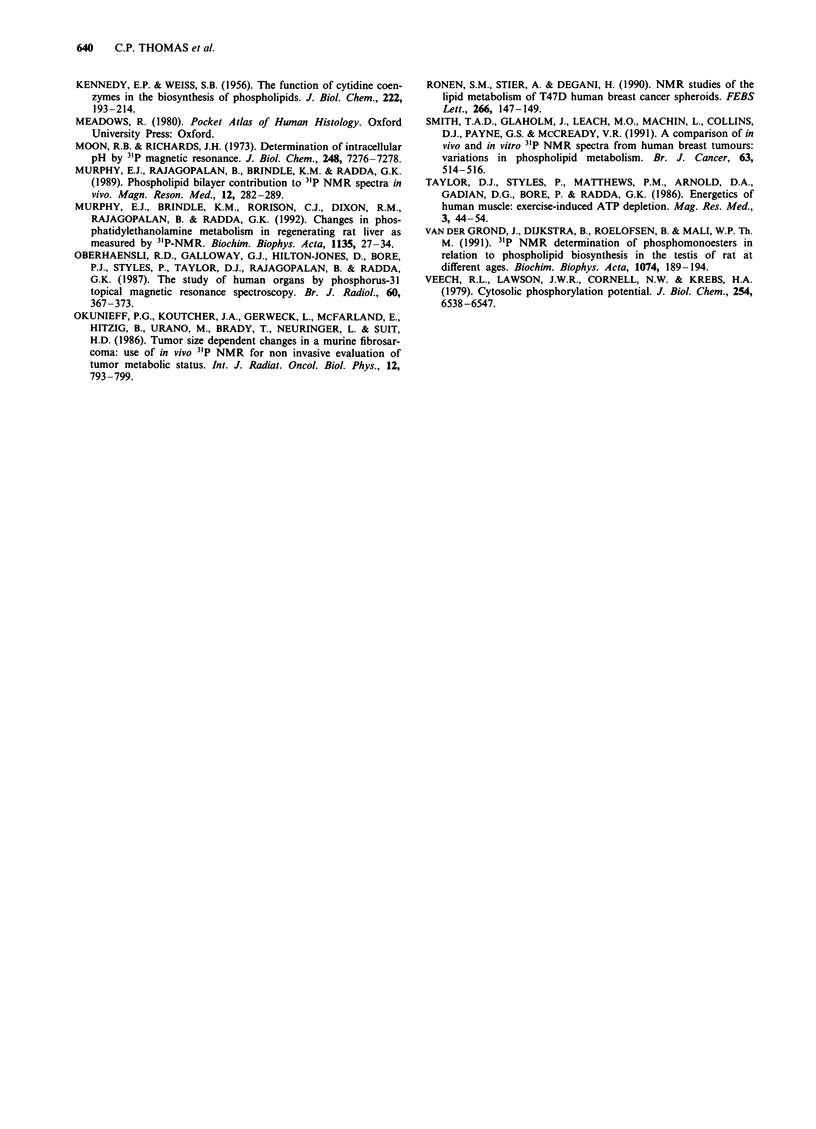# Phosphorus metabolism during growth of lymphoma in mouse liver: a comparison of 31P magnetic resonance spectroscopy in vivo and in vitro.

**DOI:** 10.1038/bjc.1994.124

**Published:** 1994-04

**Authors:** C. P. Thomas, R. M. Dixon, M. Tian, S. A. Butler, C. J. Counsell, J. K. Bradley, G. E. Adams, G. K. Radda

**Affiliations:** Department of Biochemistry, University of Oxford, UK.

## Abstract

**Images:**


*Br. J. Cancer* (1994), **69**, 633–640 © Macmillan Press Ltd., 1994

### Phosphorus metabolism during growth of lymphoma in mouse liver: a comparison of ³¹P magnetic resonance spectroscopy *in vivo* and *in vitro**

C.P. Thomas[1], R.M. Dixon[1], M. Tian[1], S.A. Butler[2], C.J.R. Counsell[2]†, J.K. Bradley[2], G.E. Adams[2] & G.K. Radda[1]

[1]*MRC Biochemical and Clinical Magnetic Resonance Unit, Department of Biochemistry, University of Oxford, South Parks Road, Oxford OX1 3QU, UK;* [2]*MRC Radiobiology Unit, Chilton, Didcot OX11 0RD, UK.*

**Summary** Large phosphomonoester (PME) signals are detected in the phosphorus magnetic resonance spectra ($^{31}P$ MRS) of many neoplastic and rapidly dividing tissues. In addition, alterations in phosphodiester (PDE) signals are sometimes seen. The present study of a murine lymphoma growing in liver showed a positive correlation between the hepatic PME/PDE ratio measured *in vivo* by $^{31}P$ MRS at 4.7 T and the degree of lymphomatous infiltration in the liver, quantified by histology. High-resolution $^{31}P$ MRS of liver extracts at 9.7 T showed that the PME peak consists largely of phosphoethanolamine (PE) and to a lesser extent of phosphocholine (PC). The concentration of both PE and PC increased positively with lymphomatous infiltration of the liver. *In vivo*, the PDE peak contains signals from phospholipids (mostly phosphatidylethanolamine and phosphatidylcholine) and the phospholipid breakdown products glycerophosphoethanolamine (GPE) and glycerophosphocholine (GPC). Low levels of GPE and GPC were detected in the aqueous extracts of the control and infiltrated livers; their concentrations remained unchanged as the infiltration increased. The total concentration of phospholipids measured by $^{31}P$ MRS of organic extracts decreased about 3-fold as the infiltration increased to 70%. Thus, our data showed that the increased PME/PDE ratio *in vivo* is due to both an increase in the PME metabolites and a decrease in the PDE metabolites. We propose that this ratio can be used as a non-invasive measure of the degree of lymphomatous infiltration *in vivo*.

The level of phosphomonoester (PME) in human tumour spectra is 2- to 4-fold higher than that obtained in the spectra of the normal tissue of origin (for a review, see Daly & Cohen, 1989). In particular, examination by $^{31}P$ magnetic resonance spectroscopy (MRS) of the livers of patients with hepatic lymphoma has shown that their spectra contain a high PME/adenosine 5′-triphosphate (ATP) ratio compared with spectra obtained from normal liver (Dixon *et al.*, 1991). Using a murine T-cell lymphoma (A120), it was found recently that the PME signal in the lymphomatous liver consists largely of phosphoethanolamine (PE), which is an intermediate of phospholipid metabolism; furthermore, this study showed that the concentration of PE measured by high-resolution $^{31}P$ MRS of liver extracts *in vitro* increased significantly with the degree of lymphomatous infiltration in the mouse liver assessed by quantitative histology (Dixon & Tian, 1993). The purpose of our project was to determine whether this phenomenon is detectable *in vivo*. Changes in steady-state cellular energetics and cellular intermediates of phospholipid metabolism during growth of another T-cell lymphoma (A55) in mouse liver were measured using *in vivo* $^{31}P$ MRS. The degree of lymphomatous infiltration in liver was measured by quantitative histology to determine whether the changes in phosphorus-containing metabolite ratios with growth of the lymphoma in liver might arise from the lymphoma cells themselves or from other cells in the environment of the tumour. Finally, to investigate further the changes in the ratios of intermediates of phospholipid metabolism during growth of lymphoma cells in liver, spectra obtained *in vivo* were compared with spectra obtained *in vitro* by high-resolution $^{31}P$ MRS of lymphomatous liver and normal liver extracts.

Correspondence: C.P. Thomas, Unité INSERM 218, Centre Léon Bérard, 28 rue Laënnec, 69008 Lyon, France.
*This work was presented at the 17th L.H. Gray Conference: Tumour assessment and response to therapy studied by MRS, 13–16 April 1992, Canterbury, UK, and at the XIIème Forum de Cancérologie, 11–13 June 1992, Paris, France.
†Present address: Medical Research Council, 20 Park Crescent, London W1N 4AL, UK.
Received 2 June 1993; and in revised form 29 October 1993.

### Materials and methods

### Mice, tumour model and experimental design

Eight- to-twelve-week-old male CBA/H mice were injected intravenously (i.v.) with $10^5$ cells from a T-cell lymphoblastic lymphoma (A55). Additional mice of the same age were not injected, and were kept under identical conditions as controls. The cell line was established from a tumour which arose after gamma-irradiation (Cobb & Butler, 1986). After injection of tumour cells, livers of six control and 35 treated mice were examined *in situ* by $^{31}P$ MRS between 1 and 25 days following injection. Mice were anaesthetised before the MRS experiment with a 1:1:2 mixture of Hypnorm (fentanyl–flunanisone)–Hypnovel (midazolam)–water at an i.p. dose of 0.15 ml per mouse. Water at 37°C, circulating through a jacket surrounding the mouse, helped to maintain its body temperature while in the magnet. At the end of each MRS investigation, the mouse was sacrificed by cervical dislocation, and the liver was removed immediately after the death of the animal. Each sample was weighed and quickly divided into two portions. One portion was frozen in liquid nitrogen for high-resolution $^{31}P$ MRS of extracts *in vitro*. The other portion was kept in formol saline for histology. Experimental procedures and care of animals were in accordance with the Animals Scientific Procedures Act 1986 (UK) and the associated 'Code of practice for the housing and care of animals used in scientific procedures' (HMSO, 1989).

### *³¹P MRS* in vivo

Anaesthetised mice were placed in a 4.7 T, 30 cm horizontal bore superconducting magnet (Oxford Instruments) interfaced to a SISCO 200 spectrometer. A two-turn, 10-mm-diameter surface coil was placed under the liver with the mouse positioned on its anterior side. The surface coil was placed below the diaphragm of the mouse. Attention was paid to ensure that the surface coil was always positioned at the same place with respect to the liver during the set of experiments. The liver lies against the ventral body wall of the mouse, behind a layer of muscle that is approximately 1 mm thick. In the control mouse, the liver extends to a depth of about 4 mm from the surface. Proton imaging was used to verify that the sensitive region of the coil did not extend below this depth, and therefore that signals from deeper lying tissues were not detected (results not shown). The magnetic field homogeneity was adjusted by observing the proton signal from tissue water. The line width of the water signal was about 70–100 Hz. Phosphorus-31 spectra of liver were obtained at a frequency of 80 MHz and from 256 pulses at a repetition rate of 2 s, i.e. spectra were acquired in about 8 min. These parameters give almost completely relaxed resonances for hepatic ATP (Oberhaensli *et al.*, 1987). Other signals were not corrected for partial saturation effects. Since recent evidence suggested that the longitudinal relaxation time ($T_1$) of the PME increased with the proportion of phosphoethanolamine (Cox *et al.*, 1991), this would mean that under the conditions of the study PME at high levels of infiltration would tend to be underestimated, and this, if anything, decreased the significance of the results. The peak area of each major line in the $^{31}P$ spectrum was calculated after baseline correction.

The spectra were fitted using an in-house program (C.J.R. Counsell, MRC). It is interactive, with optimisation of a manually fitted spectrum. The procedure begins by identifying points on a baseline. The lines in the spectrum are then fitted approximately with Lorentzian-shaped lines having four adjustable parameters (frequency, line width, amplitude, phase). The fitted spectrum is used as a starting point from which to minimise the chi-squared parameter. The baseline is optimised by adding Chebyscheff polynomials. It was possible to distinguish seven major lines in the phosphorus spectrum [nucleotide triphosphate (NTP)-α,-β and -γ, PME, PDE, inorganic phosphate ($P_i$) and phosphocreatine (PCr)]. Contamination from muscle could be detected by the presence of PCr, a metabolite absent in liver. Although the $^{31}P$ pulse width was adjusted to minimise the signal from PCr in muscle, the spectrum from liver was nevertheless contaminated by signal arising from overlying muscle. In order to correct for this, spectra from leg muscle were obtained with the same repetition rate as the liver spectra, and processed identically. The measured muscle NTP-γ/PCr ratios were used to correct the liver NTP-γ peak area, based on the amount of PCr in each liver spectrum. It was found that the NTP-γ/PCr ratio in muscle was 0.41, therefore NTP-γ contribution from muscle in each liver spectrum was subtracted in the data presented in Figures 4 and 5. Since this method has been recently reported (Jehenson, 1992) and the NTP-γ/PCr ratio in human muscle does not alter with depth, and rather little between different sites (Dunn *et al.*, 1992), we consider that the method of adjusting for muscle contamination has been validated. NTP-γ was used as a measure of ATP as NTP-α was contaminated by other metabolites such as uridine diphosphoglucose (UDPG), nicotinamide adenine dinucleotide phosphate (NADP) and nucleotide diphosphate (NDP). NTP-β peak areas were very scattered. The scatter in the NTP-β values could be due to off-resonance effects, although this is unlikely at the short pulse lengths used in the study. The NTP-γ signal also contains contributions from free NDP, but since this is thought to be about 60 μM in the liver (Veech *et al.*, 1979; Brosnan *et al.*, 1990), no further correction was required. The intracellular pH of the control and lymphomatous livers ($pH_{MRS}$) was calculated from the chemical shift of the $P_i$ resonance using the equation:

$$pH_{MRS} = 6.75 + \log\ [(S - 3.27)/(5.69 - S)]$$

where $S$ is the measured chemical shift in p.p.m. of $P_i$ from muscle PCr in the $^{31}P$ spectra (Moon & Richards, 1973; Taylor *et al.*, 1986). The peaks of the spectrum were effectively deconvoluted by fitting the whole spectrum to a sum of Lorentzians, so that the 'true' frequencies of the lines were used to calculate the pH. The energy status of the lymphomatous liver was measured from the NTP-γ/$P_i$ ratio and $pH_{MRS}$. The PME and PDE signals, which both contained intermediates of phospholipid metabolism, were used to indicate the lipid metabolic status of the lymphomatous liver. This was assessed from the PME/NTP-γ, PDE/NTP-γ and PME/PDE ratios.

*High-resolution $^{31}P$ MRS of liver extracts* in vitro

*Water-soluble extracts* The method has been published previously (Dixon *et al.*, 1991). Briefly, the frozen lymphomatous and control livers were ground to a powder in a mortar placed on dry ice and cooled under liquid nitrogen. The powder was added to an ice-cold perchloric acid solution [6% (v/v), 12 ml per sample]. The mixture was homogenised (Polytron, 15 s), centrifuged for 3 min at 3,000 r.p.m. and the supernatant was neutralised with 5 M potassium hydroxide and adjusted to pH 8–9. The pellet was retained for organic extraction as described below. After 1 h on ice, the precipitated potassium perchlorate was removed by centrifugation, and the supernatant was freeze dried. Dried samples were stored at − 20°C until the MRS study. At that time, the solid samples were dissolved in 3.5 ml of a solution containing 20 mM EDTA and 15 mM Tris; the solution was then filtered through a Chelex column and finally readjusted to pH 8.5.

*Organic extracts* A modification of the method of Bligh and Dyer (1959) was used. Briefly, the pellet remaining after perchloric acid extraction of the sample was washed twice with distilled water and extracted as follows. Chloroform–methanol (2:5, 3 ml per g of tissue) and ammonium bicarbonate (1 M, 1 ml per g of tissue) were added to the pellet, which was then homogenised for 30 s with the Polytron and left on ice for 30 min. Chloroform (0.2 ml per g of tissue) and ammonium bicarbonate (2 M, 0.1 ml per g of tissue) were added to the sample and the aqueous phase was discarded after centrifugation (15 min, 3,000 r.p.m.). Finally, chloroform–methanol (2:5, 15 ml per sample) was added to the organic layer and the sample was recentrifuged (3,000 r.p.m., 5 min). The supernatant was collected, the solvent was evaporated and the sample was stored at − 20°C until the MRS study. At that time, the dry sample was redissolved in 3.5 ml of a solution containing 5% (v/v) cholate and 50 mM EDTA, pH 8, and filtered before use.

*$^{31}P$ MRS of the extracts* $^{31}P$ nuclear magnetic resonance spectra were obtained on a 9.5 T magnet interfaced to a Bruker AM400 spectrometer. The probe consisted of a 12-mm-diameter phosphorus coil surrounded by a separate coil tuned to the proton frequency. Spectra were obtained at room temperature at a frequency of 160 MHz for $^{31}P$. Spectra of aqueous extracts were acquired with a pulse angle of 60° with an interpulse delay of 11 s and were collected with a spectral width of 8,000 Hz in 16K data points. All spectra were proton decoupled during acquisition. Decoupling was gated off during the relaxation delay. An exponential line broadening of 2 Hz was applied to the free induction decay before Fourier transformation. Glycerophosphocholine (GPC) was used an an internal chemical shift reference (2.9 p.p.m. relative to PCr at 0 p.p.m.). Spectra of organic extracts were obtained with the same acquisition parameters as used for the aqueous extracts, except that they were collected with a spectral width of 6,000 Hz in 8K data points. A coaxial capillary containing methylene diphosphonate (MDP) acted as a concentration and chemical shift standard. The MDP resonance was set to 19 p.p.m. relative to PCr at 0 p.p.m.

*Histology*

The livers were fixed in 10% formol saline and stained with haematoxylin and eosin. The livers were processed to paraffin blocks and a central section of 2 μm was cut. The degree of lymphomatous infiltration in liver was assessed using an ocular grid to measure the surface occupied by the lymphoma cells relative to the total surface area. The amounts of macrophage infiltration and necrosis during growth of the lymphoma in liver were also estimated.

### *Statistics*

Numerical results are quoted as mean ± s.d. Correlation coefficients were determined using the non-parametric Spearman rank-order correlation test.

### Results

### *Progression of the disease*

At the time of the MRS experiment, the average weight of the mice was 29 ± 2 g ($n$ = 10) for the control group and 28 ± 2 g ($n$ = 79) for the lymphoma-bearing group whatever the stage of the disease. The first histopathological evidence of disease was the invasion of the spleen at 6 days after injection of lymphoma cells. The liver started to increase in weight from day 17 after the injection of lymphoma cells; thereafter the volume doubling time was 5 days until sacrifice of the animal on about day 25 (Figure 1a). At the histological level, no lymphomatous infiltration of the liver could be observed 6 days after injection of the A55 cells (Figure 2a). Lymphomatous infiltration of the liver could be first detected on day 12; at that time, the lymphomatous infiltration was about 5% (Figure 2b). At day 18, the degree of lymphomatous infiltration in the liver was about 25% (Figure 2c). At day 19, when lymphoma cells made up about 30% of the liver, necrotic areas were detected. From days 19 to 25, the necrotic areas did not have a heavy infiltrate of macrophages and did not involve more than 10% of the liver mass. At day 24, animals began to die when the lymphomatous infiltration of liver reached about 70% (Figure 2d). At all stages of the disease, the amount of macrophage and lymphocyte infiltration was certainly less than 5% and probably less than 2%. A strong positive linear correlation between degree of lymphomatous infiltration in liver as measured by histology and liver weight was found [$r = 0.94$ ($n$ = 20), $P < 0.001$] (Figure 1b). These findings are consistent with the increase in liver weight during growth of lymphoma cells being due largely to the increase in lymphoma cell numbers.

**Figure 1** **a**, Increase in weight of the lymphomatous liver with days after intravenous injection of the A55 lymphoma cells in mice. Hatched areas show the control liver and their standard deviation. **b**, Significant increase in weight of the lymphomatous liver with degree of infiltration: $r = 0.94$ ($n = 20$), $P < 0.001$.

### *$^{31}$P MRS* in vivo

Livers of six control mice and 35 lymphomatous mice were examined *in situ* by $^{31}$P MRS. Changes in phosphorus spectra of the livers of mice injected with the A55 lymphoma cells are shown in Figure 3. Spectra obtained at day 14, when infiltration of the liver had reached about 5%, were similar to control spectra; at day 24, the lymphomatous infiltration in the liver was about 70% and major changes in phosphorus metabolism were observed: PME and $P_i$ signals increased and the $P_i$ resonance shifted to the right, demonstrating cellular acidification. However, Figure 3 shows that NTP metabolites appeared to remain stable with growth of lymphoma cells in liver. Consequently, although $P_i$ increased in the very late stages of lymphomatous infiltration, no significant correlation was detected between the NTP-$\gamma$/$P_i$ ratio and the degree of infiltration ($r = -0.005$, $P > 0.1$) (Figure 4a). However, a significant negative correlation exists between $pH_{MRS}$ and the degree of infiltration ($r = -0.68$, $P < 0.001$) (Figure 4b).

A poor, non-significant correlation was found between the PME/NTP-$\gamma$ ratio and the degree of lymphomatous infiltration in the liver ($r = 0.3$, $0.05 < P < 0.1$) (Figure 5a). No significant correlation was observed between the PDE/NTP-$\gamma$ ratio and the degree of infiltration ($r = -0.23$, $P > 0.1$) (Figure 5b). The PME/PDE ratio correlated positively with the degree of lymphomatous infiltration ($r = 0.54$, $0.01 < P < 0.001$).

### *$^{31}$P MRS of liver extracts* in vitro

The liver extracts of three control mice and 18 lymphomatous mice were examined by $^{31}$P MRS *in vitro.*

*Aqueous extracts* The phosphomonoester region of aqueous extracts of normal mouse liver contained mostly glycerolphosphate (GP) and adenosine 5′-monophosphate (AMP), although these increase rapidly after excision of the liver, and may not reflect the concentration *in vivo*; phosphoethanolamine (PE) and phosphocholine (PC) were also identified (Figure 6a). These metabolites increased in the lymphomatous liver (Figure 6b). Indeed, Figure 7a and b shows that the ratio of PE and PC to total acid-extractable phosphate (PE/Tot P and PC/Tot P) both increased significantly with lymphomatous infiltration in liver ($r = 0.89$, $P < 0.001$, and $r = 0.65$, $P < 0.005$, respectively). However, PE/Tot P increased faster than PC/Tot P, reaching about ten times its control level, while PC/Tot P only doubled at 80% infiltration (Figure 7a and b). The other major phosphomonoesters (AMP and GP) decreased as the lymphomatous infiltration in liver increased; as PE and PC both increased and AMP and GP both decreased with infiltration, the total PME/Tot P ratio *in vitro* remained unchanged as invasion of lymphoma cells increased (results not shown).

The phosphodiester region of aqueous exracts of normal mouse liver is composed of glycerophosphoethanolamine (GPE) and glycerophosphocholine (GPC), which are degradation products of phospholipids; these two metabolites did not change in the lymphomatous liver (Figure 6). Indeed, the ratio of water-soluble PDE to total acid-extractable phosphate did not alter significantly with infiltration of the lymphoma in liver ($P > 0.1$) (data not shown).

*Organic extracts* The $^{31}$P nuclear magnetic resonance spectra of organic extracts of the normal and lymphomatous mouse liver are shown in Figure 8. Major phospholipids such as phosphatidylethanolamine (PtdE) and phosphatidylcholine (PtdC) were observed, but other metabolites such as phosphatidylinositol, phosphatidylserine, sphingomyelin and cardiolipin were also identified. The total concentration of phospholipid per gram wet weight decreased to about a third of

Figure 2 The bar corresponds to 100 μm. **a**, Mouse liver 6 days after injection of lymphoma cells. No infiltration is visible. Although small numbers of A55 cells may be present they cannot be distinguished from the uniformly distributed hepatocytes which predominate. Asterisk, centrilobular vein; arrow, portal vein, **b**, Twelve days after injection of lymphoma cells. Areas of lymphoma can be seen (arrowheads), mostly surrounding the central vein (asterisk). The area of lymphomatous infiltration is 5%. **c**, Eighteen days after injection of lymphoma cells. Large areas of lymphoma (arrowheads) invade the liver parenchyma. The area of lymphomatous infiltration is 20%. **d**, Twenty-four days after injection of lymphoma cells. Liver is almost completely replaced by lymphoma. Areas of necrosis are present (stars). The area of lymphomatous infiltration is 70%. The area of necrosis is 20%.

**Figure 3** Changes in $^{31}P$ nuclear magnetic resonance spectra of mouse liver with degree of lymphoma infiltration. PME, phosphomonoesters; $P_i$, inorganic phosphate; PDE, phosphodiesters; PCr, phosphocreatine; NTP-α,-β,-γ = nucleoside triphosphates.

its control level as the infiltration increased [$r = -0.81$ ($n = 16$), $P < 0.001$], but the ratios of the various phospholipids were similar in the lymphomatous and control livers [i.e. PtdE/PtdC = $0.48 \pm 0.09$ ($n = 16$) for the lymphomatous liver *vs* $0.52 \pm 0.02$ ($n = 2$) for controls].

### Discussion

The present study shows that a positive correlation exists between the hepatic PME/PDE ratio measured *in vivo* by $^{31}P$ MRS and the degree of lymphomatous infiltration in the liver, quantified by histology (Figure 5c). Since the number of normal lymphocytes and macrophages is very low in the infiltrated liver, we emphasise that the increased PME/PDE ratio is largely due to the increase in lymphoma cell number. To investigate further the changes underlying the increased PME/PDE ratio *in vivo*, high-resolution $^{31}P$ MRS of extracts

**Figure 4** Steady-state cellular phosphorus energy metabolism parameters (NTP-$\gamma$/$P_i$ ratio **a** and $pH_{MRS}$ **b**) measured from $^{31}$P nuclear magnetic resonance spectra, such as those shown in Figure 3, as a function of the lymphoma infiltration in mouse liver. Hatched areas show the parameters of 11 control livers and their standard deviation; those of 35 lymphomatous livers are represented by the open symbols (□). A correlation exists between $pH_{MRS}$ and infiltration ($r = -0.68$, $P < 0.001$).

**Figure 5** Steady-state cellular intermediates of phospholipid metabolism parameters (PME/NTP-$\gamma$ **a**, PDE/NTP-$\gamma$ **b** and PME/PDE **c**) measured from $^{31}$P nuclear magnetic resonance spectra, such as those shown in Figure 3, as a function of the lymphoma infiltration in mouse liver. Hatched areas show the parameters of 11 control livers and their standard deviation; those of 35 lymphomatous livers are represented by the open symbols (□). A correlation exists between the PME/PDE ratio and infiltration ($r = 0.54$, $P < 0.001$).

was performed *in vitro* at 9.5 T. This showed that the PME increase was largely due to an increase in PE and to a lesser extent to PC (Figure 7). In another mouse lymphoma (A120), PE also correlated positively with the degree of lymphomatous infiltration in the liver while PC remained unchanged (Dixon & Tian, 1993).

The PDE peak contains signals from products of phospholipid breakdown (GPE and GPC), but the ratio of PDE to total acid-extractable phosphate (PDE/Tot P) remained unchanged with the degree of lymphomatous infiltration in the liver. However, the PDE/Tot P ratio obtained *in vitro* (less than 5%) is not similar to the PDE/Tot P ratio obtained *in vivo* (around 20%), suggesting that other metabolites (e.g. membrane phospholipids such as phosphatidylethanolamine and phosphatidylcholine) contribute to the peak *in vivo*; the same phenomenon was also observed by Smith *et al.* (1991) in breast tumours. Phospholipids were measured by $^{31}$P MRS of organic extracts, which showed that their total concentration decreased as the infiltration of the lymphoma in the liver increased. This result is probably due to the smaller amount of endoplasmic reticulum in lymphoma cells compared with hepatocytes, as can be seen from electron microscopy of the two types of cell (e.g. Meadows, 1980). We could speculate why the phospholipid concentration in extracts decreased by about 3-fold as the infiltration increased to 70%, while the PDE peak *in vivo* (relative to NTP) decreased only by about 30% (Figure 5b), and this decrease did not reach statistical significance. The hepatic phosphodiester peak *in vivo* at 1.9 T was shown to consist largely of membrane phospholipids, the signal being broadened as a result of chemical shift anisotropy (Murphy *et al.*, 1989). This broadening is field dependent, and only a small proportion of the total phospholipid is likely to be detected in the relatively narrow component that is quantifiable in the spectrum *in vivo* at the magnetic field strength used in the present study (4.7 T). This peak may also contain signals from water-soluble PDEs (i.e. GPE and GPC), nucleic acid and non-bilayer phospholipids, although the relative proportions of these are unknown. Phospholipid bilayer structures are disrupted by extraction with chloroform and methanol, therefore our measurement of total phospholipid concentration in extracts was quantitative. Thus, it may be that the PDE peak measured *in vivo* decreased less than the total phospholipid concentration, if phospholipid constituted only a proportion of the peak *in vivo* at this magnetic field, and if other components remained constant. Finally, the relative ratios of the various phospholipids showed no significant correlation with the degree of lymphomatous infiltration in the liver. Similar results were obtained with another murine lymphoma (A120) (Dixon & Tian, 1993) and with breast tumour spheroids (Ronen *et al.*, 1990) which confirmed that the composition of phospholipid membranes is highly regulated even in the presence of a high concentration of PE.

The increase in the PME/PDE ratio *in vivo* with lymphomatous infiltration was not as large as that predicted from the increase in the concentration of hepatic PE and decrease in total phospholipids measured in extracts. A likely explanation for this is that only a proportion of the PME signal *in vivo* is PE, and that the PDE signal *in vivo* is not composed only of phospholipids. Constant levels of other components of these signals will tend to mask changes in the PME/PDE ratio. Thus it can be seen that metabolite concen-

**Figure 6** $^{31}P$ nuclear magnetic resonance spectra of aqueous extracts of mouse liver with or without lymphoma. PE, phosphoethanolamine; PC, phosphocholine; α-GP, glycerol phosphate; AMP, adenosine monophosphate; GPE, glycerophosphoethanolamine; GPC, glycerophosphocholine.

**Figure 7** Hepatic phosphoethanolamine and phosphocholine as a percentage of total acid-extractable phosphate, measured from $^{31}P$ nuclear magnetic resonance spectra of aqueous extracts, such as those shown in Figure 6, as a function of the degree of A55 lymphoma infiltration in mouse liver. Control liver ($n = 3$) (■) and lymphomatous liver ($n = 18$) (□). Correlations exist between both PE/Tot P and PC/Tot P and lymphoma infiltration ($r = 0.89$, $P < 0.001$, and $r = 0.65$, $P < 0.01$, respectively).

**Figure 8** $^{31}P$ nuclear magnetic resonance spectra of organic extracts of mouse liver with and without A55 lymphoma. PtdC, phosphatidylcholine; PtdI, phosphatidylinositol; PtdS, phosphatidylserine; PtdE, phosphatidylethanolamine; Sph, sphingomyelin; CL, cardiolipin.

trations measured *in vitro* must be used with caution to predict or interpret spectral changes *in vivo*.

The role of phosphoethanolamine in tumour cells is not well understood. It has been suggested that high levels of PE and PC reflect rapid phospholipid synthesis, since they are intermediates of the synthetic pathways from ethanolamine and choline to their phospholipids via CDP-ethanolamine or CDP-choline (Kennedy & Weiss, 1956). There is indirect evidence for this:

- PE and PC levels increased 2-fold in exponentially growing human MCF-7 breast tumour cells compared with confluent cells (Daly *et al.*, 1987).
- In the developing rat testis, the rates of PtdE and PtdC synthesis correlated with the size of the PME/ATP ratio measured by $^{31}P$ MRS (Van der Ground *et al.*, 1991).
- In the regenerating rat liver, increases in the rates of both PtdE and PtdC were observed (Houweling *et al.*, 1991, 1992), and the concentration of PE, but not of PC, was elevated compared with that measured in sham-operated controls (Murphy *et al.*, 1992).

In contrast, it was recently shown that PtdE synthesis is actually slower in mouse livers infiltrated by a murine lymphoma (similar to the one used in the present study) than in normal mouse liver, despite the hepatic PE concentration being significantly increased (Dixon & Tian, 1993). Thus, high levels of PE and PC do not necessarily reflect rapid phospholipid synthesis. An alternative explanation is that there is a high rate of phospholipid breakdown in the tumour cells, either by phospholipase C, leading to PE or PC and diacylglycerol, or by phospholipase D, leading to ethanolamine or choline and phosphatidic acid. This breakdown may be related to abnormal signalling pathways in tumour cells (since diacylglycerol and phosphatidic acid are both second messengers involved in signal transduction) similar to the well-known phosphoinositide pathways (reviewed by Berridge, 1984). There is evidence for PtdC breakdown being important in the production of diacylglycerol (Exton, 1990). Murphy *et al.* (1992) reported that the regenerating rat liver showed an elevation in diacylglycerol that had a similar time course to the increase in PE, although another study found no significant change in hepatic diacylglycerol 22 h after partial hepatectomy (Houweling *et al.*, 1992). Evidence for phospholipid breakdown being important in signalling pathways in tumour cells is as yet lacking, but could provide an explanation for the widespread findings of high levels of PE and PC in these cells.

The present study of a murine lymphoma growing in liver showed that no correlation exists between the NTP-$\gamma$/$P_i$ ratio measured *in vivo* by $^{31}$P MRS and the degree of lymphomatous infiltration in liver, quantified by histology (Figure 4a). This result, which is in agreement with those observed in the literature (e.g. Okunieff *et al.*, 1986), indicates that $^{31}$P MRS is probably not able to detect, at the moment, *small* changes in phosphorus energy metabolism parameters in very well-vascularised tumour tissue such as that in early infiltrating liver; this may not be so in other poorly vascularised solid tumours in which hypoxia appears to develop at an early stage. In contrast, *large* changes in phosphorus energy metabolism parameters are easily detectable by $^{31}$P MRS after chemo-and/or radiotherapies (e.g. Allavena *et al.*, 1991) or physiological manipulation of the tumour (e.g. Adams *et al.*, 1992). It is interesting to note that even appearance of necrotic areas did not influence the NTP-$\gamma$/$P_i$ ratio significantly, thus confirming in an animals model that necrosis seems 'invisible' to $^{31}$P MRS as suggested by Freyer *et al.* (1991) on a spheroid model *in vitro*. As suggested by these authors, it implies that in tumour chronically nutrient-deprived cells which should be close to necrotic areas would not contribute significantly to the NTP-$\gamma$/$P_i$ ratio. The large increase in $P_i$ seen in the later stages of lymphomatous infiltration in liver (Figure 3) probably therefore arises from acutely nutrient-deprived cells as a result of intermittent changes in tumour blood flow. Such a phenomenon has been demonstrated in this mouse lymphoma (A.I. Minchinton & L.M. Cobb, personal communication). Necrosis probably contributes to the increased acidification of the lymphomatous liver, as a significant negative correlation exists between $pH_{MRS}$ and degree of lymphomatous infiltration in liver (Figure 4b). In contrast, in a well-vascularised tumour without necrosis, no change in $pH_{MRS}$ was observed during growth (Allavena *et al.*, 1991).

In summary, the present study has shown using $^{31}$P MRS *in vivo* that the PME/PDE metabolite ratio correlated positively with the degree of lymphomatous infiltration in mouse liver. Although, the statistical significance of the increase of the PME/PDE ratio *in vivo* was relatively low, the changes were consistent with the results from the extract studies. The *in vitro* study showed that the increase in the PME/PDE ratio observed *in vivo* was due to both an increase in the PME metabolites and a decrease in the PDE metabolites. The PME increase was largely due to an increase in phosphoethanolamine and the PDE decrease was due to a decrease in total phospholipids and not in water-soluble PDEs such as GPC and GPE. Since PEs and phospholipids comprise only a proportion of the *in vivo* PME and PDE signals, respectively, the change in the ratio of PME/PDE *in vivo* was less than that predicted from the results *in vitro*. The approximately 2-fold increase in the PME/PDE ratio in the mouse lymphomatous liver compared with normal liver was consistent with results obtained in human lymphoma (for reviews see Daly & Cohen, 1989). Thus, we suggest that the PME/PDE ratio could be a useful non-invasive measure of the degree of lymphomatous infiltration *in vivo*.

We thank Paul Bates and his team for animal care; Terry Hacker and his team for preparation of the histology slides; Leon Cobb for providing the A55 lymphoma model and for helpful advice concerning the histological quantification; Bheeshma Rajagopalan, Ian Stratford and Pauline Wood for continuous support and enthusiasm. This work was supported in part by the Imperial Cancer Research Fund.

### References

ADAMS, G.E., BREMNER, J.C.M., COUNSELL, C.J.R., STRATFORD, I.J., THOMAS, C.P. & WOOD, P.J. (1992). Magnetic resonance spectroscopy studies on experimental murine and human tumours: comparison of changes in phosphorus metabolism with induced changes in vascular volume. *Int. J. Radiat. Oncol. Biol. Phys.*, **22**, 467–471.

ALLAVENA, C., GUERQUIN-KERN, J.L. & LHOSTE, J.M. (1991). Follow up by $^{31}$P NMR spectroscopy of the energy metabolism of malignant tumor in rats during treatment. *Radiother. Oncol.*, **21**, 48–52.

BERRIDGE, M.J. (1984). Inositol triphosphate and diacylglycerol as second messengers. *Biochem. J.*, **220**, 345–360.

BLIGH, E.G. & DYER, W.J. (1959). A rapid method of total lipid extraction and purification. *Can. J. Biochem. Physiol.*, **37**, 911–917.

BROSNAN, M.J., CHEN, L., VAN DYKE, T.A. & KORETSKY, A.P. (1990). Free ADP levels in transgenic mouse liver expressing creatine kinase. *J. Biol. Chem.*, **265**, 20849–20855.

COBB, L.M. & BUTLER, S.A. (1986). The influence of prior total body irradiation on the tissue distribution of mouse lymphoma/leukemia. *Int. J. Radiat. Oncol. Biol. Phys.*, **12**, 83–88.

COX, I.J., COUTTS, G.A., GADIAN, D.G., GHOSH, P., SARGENTONI, J. & YOUNG, I.R. (1991). Saturation effects in phosphorus-31 magnetic resonance spectra of the human liver. *Magn. Res. Med.*, **17**, 53–61.

DALY, P.F. & COHEN, J.S. (1989). Magnetic resonance spectroscopy of tumors and potential *in vivo* clinical applications: a review. *Cancer Res.*, **49**, 770–779.

DALY, P.F., LYON, R.F., FAUSTINO, P.J. & COHEN, J.S. (1987). Phospholipid metabolism in cancer cells monitored by $^{31}$P NMR spectroscopy. *J. Biol. Chem.*, **262**, 14875–14878.

DIXON, R.M. & TIAN, M. (1993). Phospholipid synthesis in the lymphomatous mouse liver studied by $^{31}$P nuclear magnetic resonance spectroscopy and by administration of $^{14}$C-radiolabeled compounds *in vivo*. *Biochim. Biophys. Acta*, **1181**, 111–124.

DIXON, R.M., ANGUS, P.W., RAJAGOPALAN, B. & RADDA, G.K. (1991). Abnormal phosphomonoester signals in $^{31}$P MR spectra from patients with hepatic lymphoma. A possible marker of liver infiltration and response to chemotherapy. *Br. J. Cancer*, **63**, 953–958.

DUNN, J.F., KEMP, G.J. & RADDA, G.K. (1992). Depth selective quantification of phosphorus metabolites in human calf muscle. *NMR Biomed.*, **5**, 154–160.

EXTON, J.H. (1990). Signalling through phosphatidylcholine breakdown. *J. Biol. Chem.*, **265**, 1–4.

FREYER, J.P., SCHOR, P.L., JARETT, K.A., NEEMAN, M. & SILLERUD, L.O. (1991). Cellular energetics measured by phosphorus nuclear magnetic resonance spectroscopy are not correlated with chronic nutrient deficiency in multicellular tumor spheroids. *Cancer Res.*, **51**, 3831–3837.

HMSO (1989). Code of Practice for the Housing and Care of Animals used in Scientific Procedures. HMSO: London.

HOUWELING, M., TIJBURG, L.B.M., JAMIL, H., VANCE, D.E., NYATHI, C.B., VAARTJES, W.J. & VAN GOLDE, L.M.G. (1991). Phosphatidylcholine metabolism in rat liver after partial hepatectomy. *Biochem. J.*, **278**, 347–351.

HOUWELING, M., TIJBURG, L.B.M., VAARTJES, W.J. & VAN GOLDE, L.M.G. (1992). Phosphatidylethanolamine metabolism in rat liver after partial hepatectomy. *Biochem. J.*,, **283**, 55–61.

JEHENSON, P. (1992). Correcting for the contamination by muscle signal of *in vivo* $^{31}$P NMR spectra of the liver and kidney. *J. Magn. Res.*, **96**, 181–184.

KENNEDY, E.P. & WEISS, S.B. (1956). The function of cytidine coenzymes in the biosynthesis of phospholipids. *J. Biol. Chem.*, **222**, 193–214.

MEADOWS, R. (1980). *Pocket Atlas of Human Histology*. Oxford University Press: Oxford.

MOON, R.B. & RICHARDS, J.H. (1973). Determination of intracellular pH by $^{31}$P magnetic resonance. *J. Biol. Chem.*, **248**, 7276–7278.

MURPHY, E.J., RAJAGOPALAN, B., BRINDLE, K.M. & RADDA, G.K. (1989). Phospholipid bilayer contribution to $^{31}$P NMR spectra *in vivo*. *Magn. Reson. Med.*, **12**, 282–289.

MURPHY, E.J., BRINDLE, K.M., RORISON, C.J., DIXON, R.M., RAJAGOPALAN, B. & RADDA, G.K. (1992). Changes in phosphatidylethanolamine metabolism in regenerating rat liver as measured by $^{31}$P-NMR. *Biochim. Biophys. Acta*, **1135**, 27–34.

OBERHAENSLI, R.D., GALLOWAY, G.J., HILTON-JONES, D., BORE, P.J., STYLES, P., TAYLOR, D.J., RAJAGOPALAN, B. & RADDA, G.K. (1987). The study of human organs by phosphorus-31 topical magnetic resonance spectroscopy. *Br. J. Radiol.*, **60**, 367–373.

OKUNIEFF, P.G., KOUTCHER, J.A., GERWECK, L., MCFARLAND, E., HITZIG, B., URANO, M., BRADY, T., NEURINGER, L. & SUIT, H.D. (1986). Tumor size dependent changes in a murine fibrosarcoma: use of *in vivo* $^{31}$P NMR for non invasive evaluation of tumor metabolic status. *Int. J. Radiat. Oncol. Biol. Phys.*, **12**, 793–799.

RONEN, S.M., STIER, A. & DEGANI, H. (1990). NMR studies of the lipid metabolism of T47D human breast cancer spheroids. *FEBS Lett.*, **266**, 147–149.

SMITH, T.A.D., GLAHOLM, J., LEACH, M.O., MACHIN, L., COLLINS, D.J., PAYNE, G.S. & MCCREADY, V.R. (1991). A comparison of *in vivo* and *in vitro* $^{31}$P NMR spectra from human breast tumours: variations in phospholipid metabolism. *Br. J. Cancer*, **63**, 514–516.

TAYLOR, D.J., STYLES, P., MATTHEWS, P.M., ARNOLD, D.A., GADIAN, D.G., BORE, P. & RADDA, G.K. (1986). Energetics of human muscle: exercise-induced ATP depletion. *Mag. Res. Med.*, **3**, 44–54.

VAN DER GROND, J., DIJKSTRA, B., ROELOFSEN, B. & MALI, W.P. Th. M. (1991). $^{31}$P NMR determination of phosphomonoesters in relation to phospholipid biosynthesis in the testis of rat at different ages. *Biochim. Biophys. Acta*, **1074**, 189–194.

VEECH, R.L., LAWSON, J.W.R., CORNELL, N.W. & KREBS, H.A. (1979). Cytosolic phosphorylation potential. *J. Biol. Chem.*, **254**, 6538–6547.